# The impact of basic public health services on migrants’ settlement intentions

**DOI:** 10.1371/journal.pone.0276188

**Published:** 2022-10-17

**Authors:** Haowen Jia

**Affiliations:** Research Institute of Economics and Management, Southwestern University of Finance and Economics, Chengdu, Sichuan, China; Shenzhen University, CHINA

## Abstract

An important task in the urbanization process of developing countries is to promote and support migrants’ settlement in cities. Based on the China Migrants Dynamic Survey of 2018, this paper analyzes how basic public health services (BPHSs) impact migrants’ settlement intentions. This study shows that establishing health records and access to health-related knowledge significantly and positively impact migrants’ intentions to settle permanently in inflow areas by increasing their health status and degree of social integration. Yet, this paper discusses how the trends are heterogeneous, finding that BPHSs more significantly impact the settlement intentions of female, less-educated and rural migrants. The findings of this paper provide new factual evidence that may support government policymaking to further improve migrants’ utilization of medical and health resources and their intention to settle down.

## 1. Introduction

Over the past four decades, China has witnessed an unprecedented high pace of urbanization, with rural-to-urban migrants being key drivers of this process [1]. The National Bureau of Statistics for China (2020) has shown that the urbanization level in China has reached 60.65%, and the number of migrants exceeded 236 million by 2019. According to Northam’s theory on the stages of urbanization, China entered the late-intermediate stage of urbanization in 2011 [2, 3].

With the development of people-centered urbanization, a series of household registration (Hukou) reforms have occurred to promote the conversion of rural migrants to urban citizens, and migrants’ equal access to basic public services has been prioritized in China. However, achieving this goal requires not only the support and guidance of relevant government policies but also migrants’ strong intent to settle down permanently in their destination cities. In that context, understanding migrants’ settlement intent and its determinants, and monitoring changes to this, are greatly important for both academic studies and policymaking [4].

Taking China as the research background, existing literature has paid much attention to the factors impacting rural migrants’ intentions to settle permanently in urban areas. The Hukou system has long been a factor affecting rural migrants’ settlement in urban areas since it restricted rural migrants’ ability to search for jobs and use public services in cities [5, 6]. Human capital is then another factor often affecting the urban settlement intent of migrants. Studies have found that high-skilled migrants often express a stronger desire for permanent urban settlement than low-skilled migrants [7, 8]. In addition, social capital also plays an important role in determining settlement intentions. Greater social organizational participation and connections with urban locals positively impact urban settlement intentions [9, 10]. Furthermore, research has highlighted the important impact of basic medical insurance, or one of other types of social insurance, on the urban settlement intentions of migrants [11, 12]. These studies suggest the government should establish a more inclusive social welfare system to enable migrants to permanently settle in cities. Finally, some studies have noted the impact of socioeconomic factors on migrants’ settlement intentions, showing that rural migrants with a higher income and better occupational status have a stronger settlement intention [13, 14].

With the progress of urbanization and citizenization, greater attention has been paid to the medical and health services available to migrants. In recent years, thanks to financial subsidies from the Chinese government, basic public health services for migrants have been improved [15]. Today, do government-led health services encourage migrants to settle permanently in cities? Answering this question will help us to understand the important role of health services in the process of urbanization and provide policy guidance for further urbanization in developing countries.

In 2009, the Chinese government issued a landmark health policy to promote universal access to health services by launching the basic public health services (BPHSs), which aimed to provide free-of-charge essential health services for all citizens at the point of care [16]. The initial package in 2009 included nine categories: establishing health records, health education, immunization for children, health management for children, maternal health management, elderly health management, health management for residents with chronic diseases, reporting and handling infectious diseases and health management for residents with severe mental illness [17, 18].

Since the key goal of the recent healthcare reform was to provide equitable and accessible public health services to every Chinese citizen, all services in the BPHSs package were provided to all residents in the catchment area, including non-registered people (those without a local Hukou) who had lived in the inflow area for at least six months. In keeping with the policy drive toward equalization of BPHSs, the issue of access to healthcare for migrants became the focus of attention, and multiple measures were implemented by the Chinese government, accordingly, to promote access to BPHSs for migrants [16, 19].

Existing studies have proven that basic public services led by the government can improve people’s health and the overall welfare of society. For instance, the BPHSs project in China was found to be associated with improvements to maternal health services and reduced maternal mortality [20, 21]. However, the utilization rate of BPHSs by migrants remains low. The literature has speculated on the potential reasons for this from different perspectives. For example, sociocultural adaptation and social integration are highly correlated with establishing health records and providing access to health education for migrants [22, 23], and structural social capital is related to increased utilization of local public health services among migrants [24].

As described before, developing countries have invested a lot of money in providing residents with medical and health services, but the impact of these services on the process of urbanization is still unclear. This study suggests that access to BPHSs positively impacts migrants’ settlement intentions for the following two reasons. Firstly, BPHSs increase the convenience of medical and health services for migrants and also improve their health level through preventive health education, both of which significantly positively impact their health status. Existing studies have shown that, compared to those in poor health, migrants with better health tend to settle down in cities [25, 26]. Secondly, BPHSs are generally provided by community services, including health records and health-related education. When migrants receive such services, their sense of belonging to inflow areas is significantly enhanced, thus improving their social integration, which may positively impact their settlement intentions [9, 10].

Using microdata and empirical methods, this paper finds that BPHSs improve the health and social integration of migrants, thus improving their intentions to settle long-term. The main contribution of this study is its novel analysis of migrants’ intentions to settle down as impacted by the provision of public health services. This study concludes on the findings from the data collected and provides practical evidence and policy recommendations for how we can improve migrants’ utilization of medical and health resources, along with their settlement intentions.

The remainder of the paper is structured as follows: the second section describes the materials and models, the third section analyzes the empirical results and the last section concludes the paper.

## 2. Materials and methods

### 2.1. Data sources

This study used data from the China Migrants Dynamic Survey (CMDS), which was published by the National Health Commission of the People’s Republic of China in 2018. Annual report data were analyzed concerning the migrant populations of 31 provinces. At the prefecture level and county level, it used the Chinese standard administrative code and unified institutional code, the survey was conducted in accordance with random principles in the inflow areas where the migrant population was concentrated. It used a stratified, multi-stage, proportional-to-size PPS method to extract sample points according to the migrant population’s health condition and policy research needs. The respondents were migrants who had lived in the destination cities for more than one month but were not registered in the district. According to the research needs of this study, samples with missing variables (most of the missing values come from work information of respondents) were eliminated to obtain an effective research sample size of 138,854, from 31 provinces, 351 prefectures, and 1,234 districts.

### 2.2. Main variables

#### 2.2.1. Dependent variables

This study focused on the impact of basic public health services (BPHSs) on migrants’ settlement intentions. The CMDS asked whether the respondents would like to settle in inflow areas sometime soon, and then for how long the respondents would settle. According to the answers of the respondents, this paper used two variables to measure the settlement intentions of migrants: (1) settlement intention, where the value 1 indicated that the migrants would like to settle in the inflow areas soon, and 0 indicated otherwise; and (2) the length of time that the migrants would like to settle in the inflow areas, from 0 years to permanent settlement.

#### 2.2.2. Independent variables and control variables

The core independent variable was migrants’ access to basic public health services. Health services can be divided into preventive care services and therapeutic care services, but the available survey data referred only to preventive care services. To reflect the BPHSs available to migrants, this study generated an indicator variable: the establishment of health records in their community of residence, where the value 1 indicated the establishment of health records, and 0 indicated otherwise. This study selected whether respondents established health records as the definition of whether they had access to basic public health services or not, because (1) it was the first step to receiving health services, and only after the establishment of records could they receive other types of services; and (2) it was for all people, not specific groups (such as kids, maternal and old people, etc). In addition, this study concerned with migrants’ access to health-related knowledge in inflow areas in the past year, which mainly included knowledge about occupational diseases, infectious diseases, contraception and sterilization, chronic diseases, mental disorders, and public emergencies, where the value 1 indicated access to knowledge of the above items, and 0 indicated otherwise.

This study also controlled other important variables affecting migrants’ settlement intentions, which included: (1) individual characteristics of migrants, such as gender, age and age squared, marriage status, educational level, employment status, time of residence in the inflow area, health level, social insurance status and Hukou type; (2) family characteristics, such as family size, dependency ratio, household income and expenditure. Table 1 reports the definitions and descriptive statistics of the main variables used in this study.

**Table 1:** Definitions and descriptive statistics of the main variables used in this study.

Variables	Variables’ Definitions	Mean	SD
Settlement intention	Intend to settle in inflow area (= 1; otherwise = 0)	0.8582	0.3489
Settlement intention_short	Intend to settle in inflow area for 0 to 9 years (= 1; otherwise = 0)	0.2058	0.4043
Settlement intention_long	Intend to settle in inflow area for more than 10 years (= 1; otherwise = 0)	0.1034	0.3044
Settlement intention_per	Intend to settle in inflow area permanently (= 1; otherwise = 0)	0.3255	0.4686
Health record	Health record established (= 1; otherwise = 0)	0.2828	0.4504
Gender	Male (= 1; otherwise = 0)	0.5126	0.4998
Age	Age	37.4305	11.123
Educational level	High school or above (= 1; otherwise = 0)	0.4191	0.4934
Married	Married (= 1; otherwise = 0)	0.8241	0.3808
Divorced	Divorced (= 1; otherwise = 0)	0.0185	0.1349
Separated	Separated (= 1; otherwise = 0)	0.0103	0.1008
Widowed	Widowed (= 1; otherwise = 0)	0.0091	0.095
Residence time	Years of residence in inflow areas	6.7943	6.0518
Work	Have at least one job (= 1; otherwise = 0)	0.8325	0.3734
Social insurance	Attend social insurance (= 1; otherwise = 0)	0.9353	0.246
Health level	Health level (very healthy = 1; healthy = 2; unhealthy = 3; very unhealthy = 4)	1.1592	0.4271
Hukou type	Rural Hukou (= 1; otherwise = 0)	0.6838	0.465
Family size	Family population	3.1808	1.1891
Having kids	At least 1 kid under age 15 (= 1; otherwise = 0)	0.5997	0.49
Dependency ratio	Share of people under age 15 and over age 65 in the total family size	0.457	0.3954
Household income	Logarithmic annual household income	8.0987	0.6186
Household expenditure	Logarithmic annual household expenditure	8.7486	0.692

### 2.3. Model

To estimate the impact of access to the BPHSs on migrants’ settlement intentions, this study used a linear probability model, as shown in Eq (1):

$$Settlement\_intention_{i}=\alpha +\beta BPHSs_{i}+\gamma Controls_{i}+District_{d}+\varepsilon_{i,d}$$ (1)

where *Settlement_intention*_*i*_ is the settlement intention of migrant *i*, measured by the dependent variables above. *BPHSs*_*i*_ is migrant *i*‘s access to the BPHSs, including whether they have a health record and whether they can access health-related knowledge. *β* is the parameter of primary concern. If *β* is significantly positive, then access to the BPHSs positively affects the settlement intentions. *Controls*_*i*_ indicates covariates of individual and household characteristics and *District*_*d*_ is a fixed district effect. *ε*_*i*,*d*_ is a random error term. This study used Stata 16.0 to process and analyze the data. Specifically, the *reghdfe* command is used for regression analysis, which helps control for district and industry fixed effects in the regressions more easily.

It is important to note that there are limitations of the CMDS data in conducting the empirical analysis of this study. First, since this study used cross-sectional data, it was only possible to know whether the respondents had established a health record, but not the exact time when they did so. Second, the efficiency and evaluation of the access to public health services by the respondents were missing from the data, which added to the difficulty of making targeted recommendations for how to improve the public health services system. Third, limitations in the data structure prevented this paper from making causal inferences in an economic sense, so the conclusions of this paper were more akin to correlation analysis. To address the data limitations, this study minimized error confounding and obtained more accurate estimates by adding district fixed effects (as Eq (1)) to the regressions and using the instrumental variable method (see Section 3.4).

## 3. Results

### 3.1. Establishing health records and migrants’ settlement intentions

Table 2 presents the impact of establishing health records on migrants’ settlement intentions using Eq (1). After controlling individual and household characteristics, Column (3) in Table 2 shows that establishing health records significantly positively affects migrants’ settlement intentions. The intention to settle in the inflow area increased by 4.17% among those with health records compared to those without. Furthermore, Columns (4) to (6) report whether establishing health records impacted the desired length of settlement in the inflow areas in the future. Interestingly, migrants’ establishment of health records has no significant impact on their short-term settlement intentions and a significant but small impact on their long-term settlement intentions. Access to the BPHSs, meanwhile, had a significant, large, positive impact on the permanent settlement intent of migrants in inflow areas, which indicates the important role of BPHSs in improving migrants’ intentions to settle permanently in the urban area as they undergo the process of urbanization.

**Table 2:** Analysis of relationship between establishing health records and migrants’ intentions of settling.

Variables	(1)	(2)	(3)	(4)	(5)	(6)
	Settlement intention	Settlement intention	Settlement intention	Settlement intention_short	Settlement intention_long	Settlement intention_per
Health record	0.0504***	0.0427***	0.0417***	0.0016	0.0157***	0.0429***
	(0.0031)	(0.0031)	(0.0031)	(0.0035)	(0.0026)	(0.0043)
Gender		0.0078***	0.0052**	0.0298***	0.0252***	-0.0217***
		(0.0022)	(0.0022)	(0.0023)	(0.0018)	(0.0028)
Age		-0.0018***	-0.0011	-0.0002	0.0075***	-0.0112***
		(0.0007)	(0.0006)	(0.0007)	(0.0005)	(0.0009)
Age squared		0.0000	0.0000	0.0000	-0.0001***	0.0001***
		(0.0000)	(0.0000)	(0.0000)	(0.0000)	(0.0000)
Educational level		0.0309***	0.0243***	-0.0121***	-0.0039*	0.1048***
		(0.0025)	(0.0025)	(0.0029)	(0.0023)	(0.0041)
Married		0.0961***	0.0500***	-0.0131**	0.0007	0.0996***
		(0.0053)	(0.0053)	(0.0059)	(0.0035)	(0.0078)
Divorced		0.0531***	0.0219**	-0.0217**	-0.0007	0.0849***
		(0.0095)	(0.0096)	(0.0099)	(0.0069)	(0.0111)
Separated		0.0694***	0.0400***	0.0178	-0.0005	0.0577***
		(0.0110)	(0.0109)	(0.0136)	(0.0069)	(0.0139)
Widowed		0.1134***	0.0726***	-0.0396***	-0.0034	0.1665***
		(0.0128)	(0.0129)	(0.0114)	(0.0080)	(0.0152)
Residence time		0.0044***	0.0042***	-0.0063***	0.0021***	0.0117***
		(0.0002)	(0.0002)	(0.0003)	(0.0002)	(0.0003)
Work		-0.1805***	-0.1787***	-0.8876***	0.0588***	0.3112***
		(0.0106)	(0.0104)	(0.0129)	(0.0093)	(0.0161)
Social insurance		0.0282***	0.0256***	0.0236***	0.0048	-0.0107**
		(0.0057)	(0.0057)	(0.0050)	(0.0037)	(0.0053)
Health level		-0.0168***	-0.0138***	-0.0085***	-0.0016	-0.0076**
		(0.0030)	(0.0030)	(0.0029)	(0.0021)	(0.0033)
Hukou type		-0.0181***	-0.0121***	0.0238***	0.0075***	-0.0690***
		(0.0029)	(0.0029)	(0.0042)	(0.0024)	(0.0056)
Family size			-0.0004	-0.0042***	0.0020*	-0.0067***
			(0.0016)	(0.0014)	(0.0011)	(0.0021)
Having kids			-0.0088**	-0.0009	-0.0036	0.0042
			(0.0044)	(0.0045)	(0.0039)	(0.0055)
Dependency ratio			0.0455***	-0.0039	0.0310***	0.0202***
			(0.0046)	(0.0051)	(0.0044)	(0.0069)
Household expenditure			0.0179***	-0.0477***	0.0099***	0.0855***
			(0.0029)	(0.0029)	(0.0020)	(0.0038)
Household income			0.0264***	0.0077***	0.0059***	0.0282***
			(0.0024)	(0.0021)	(0.0016)	(0.0032)
District FE	Yes	Yes	Yes	Yes	Yes	Yes
N	138,854	138,854	138,854	138,854	138,854	138,854
R-squared	0.0601	0.0784	0.0835	0.1080	0.0428	0.2439

Notes: This table reports the impact of establishing health records on migrants’ settlement intentions using the OLS method in Eq (1). Work industry information and work unit information are controlled in Columns (2) to (6). Standard errors in parentheses are clustered at the district level. Significance codes

*p<0.10

**p<0.05

***p<0.01.

The results for the covariates are mostly consistent with the existing literature. Migrants with higher educational levels are more likely to intend to settle permanently in inflow areas. Compared to being unmarried, being married, divorced, separated, and widowed all have a significant positive impact on the intention to settle in inflow areas among the migrant population. At the household level, migrant families with more members, greater expenditure, and a higher combined income seem to have a higher propensity to settle for a long time in inflow areas. This study also finds that households with higher dependency ratios have higher intentions to settle in the inflow areas, especially in the long term. However, this paper does not find a significant correlation between having kids and migrants’ settlement intentions.

### 3.2. Discussion of endogenous problem

The positive impact of BPHSs on migrants’ intention to settle permanently has been demonstrated before, but the estimated results in Table 2 may be biased due to an endogenous problem, which mainly comes from reverse causality. That is to say, people who were expected to settle in the inflow areas were more likely to gain access to health services. To obtain more accurate estimation results, this paper used an instrumental variable and the 2SLS method. The instrumental variable was a dummy variable defined as below: if the average probability of establishing health records in the respondent’s community is greater than the average probability for the district, the variable is set to 1; otherwise, it is 0. The reasons for this are as follows. Firstly, BPHSs, including health records systems and health-related knowledge, are provided by communities, meaning the average probability of establishing health records represents the intensity of community health services provided. If the average probability for a community is high, then individuals in the community are more likely to receive health services. Secondly, there is currently no evidence that the provision of health services in communities can increase migrants’ intent to settle in inflow areas through channels other than improving their access to health services.

I applied the 2SLS method, and Columns (1) and (2) in Table 3 report the estimation results when using an instrumental variable according to the definition above. After excluding endogenous problems caused by reverse causality, establishing health records significantly positively impacts the settlement intent of migrants in inflow areas. In Columns (3) and (4), the definition of the instrumental variable is changed as follows: if the average probability of establishing health records in the respondent’s community is greater than the average probability for the prefecture, the variable is set to 1; otherwise, it is 0. Overall, the results in Columns (1) to (4) demonstrate a causal relationship between establishing migrants’ health records and their intent to settle.

**Table 3:** Results of our second analysis, carried out to overcome the endogenous problem.

Variables	(1)	(2)	(3)	(4)	(5)	(6)
	Settlement intention	Settlement intention_per	Settlement intention	Settlement intention_per	Settlement intention	Settlement intention_per
Health record	0.0582***	0.0613***	0.0593***	0.0714***	0.0473***	0.0612***
	(0.0115)	(0.0147)	(0.0119)	(0.0144)	(0.0072)	(0.0092)
Controls	Yes	Yes	Yes	Yes	Yes	Yes
County FE	Yes	Yes	Yes	Yes	Yes	Yes
N	138,854	138,854	138,854	138,854	18,802	18,802
R-squared	0.0235	0.0782	0.0235	0.0778	0.1300	0.2846

Notes: This table reports the impact of establishing health records on migrants’ settlement intentions when considering the endogenous problem. Columns (1) to (4) report the results of the second stage of regression using the 2SLS method, where the results of the first stage of regression are significantly positive and the F-statistics are all significantly greater than 10. Samples in Columns (5) to (6) are all with poor health (health level = 3 or 4). Controls include all covariates in Table 2. Work industry information and work unit information are controlled in all columns. Standard errors in parentheses are clustered at the district level. Significance codes

*p<0.10

**p<0.05

***p<0.01.

In Columns (5) and (6), only migrants with poor health (health level = 3 or 4) were used for the regression since they were more likely to establish health records to gain access to medical care, which partly rules out the above reverse-causality problem. The results in Table 3 show that after eliminating the bias from reverse causality, migrants’ access to BPHSs still robustly and significantly positively impacted migrants’ intentions to settle permanently.

### 3.3. Possible mechanisms

In this section, the paper discusses the mechanisms by which BPHSs affect migrants’ intent to settle down. There are two possible mechanisms, as follows. Firstly, after migrants gain access to BPHSs, the convenience of their medical care will improve, meaning their health, too, will be improved. The existing literature concludes that migrants with better health are more likely to find work in the labor market of inflow areas, which increases their likelihood of permanent settlement. Secondly, as introduced above, BPHSs are mainly provided by the community. The process of migrants using BPHSs will enhance their sense of community belonging and social integration, and thus improve their intent to settle. Columns (1) to (3) and (4) to (6) in Table 4 test the above two mechanisms, respectively.

**Table 4:** Possible mechanisms by which BPHSs affect migrants’ intent to settle down.

Variables	(1)	(2)	(3)	(4)	(5)	(6)
	Health level	Health level	Health level	Social integration	Social integration	Social integration
Health record	0.0247***	0.0264***	0.0257***	0.0699***	0.0459***	0.0597***
	(0.0034)	(0.0031)	(0.0031)	(0.0144)	(0.0072)	(0.0092)
Individual controls		Yes	Yes		Yes	Yes
Household controls			Yes			Yes
County FE	Yes	Yes	Yes	Yes	Yes	Yes
N	138,854	138,854	138,854	14,666	14,666	14,666
R-squared	0.1014	0.1864	0.1927	0.1021	0.1088	0.1119

Notes: This table reports the impact of establishing health records on migrants’ health levels and degree of social integration. Columns (4) to (6) use the 2014 wave of CMDS data for regression. Individual and household controls correspond to the variables in Table 2. Work industry information and work unit information are controlled in Columns (2) to (3) and (5) to (6). Standard errors in parentheses are clustered at the district level. Significance codes

*p<0.10

**p<0.05

***p<0.01.

After adding individual and household characteristics as control variables, the results in Column (3) of Table 4 show how establishing health records for migrants can significantly improve their health, confirming the previous hypothesis. Next, due to data limitations, CMDS data for the 2014 wave were used to examine the impact of access to BPHSs on the social integration of migrants. The social integration variable was valued from 1 to 5, as derived from respondents’ answers to the question: how much do you think you are integrated into current society? The higher the value, the greater the degree of integration of the respondent. Column (6) in Table 4 reports that, after controlling for individual and household characteristics, establishing health records positively and significantly impacts the degree of social integration, which also confirms the previous hypothesis.

### 3.4. Effects are heterogeneous at the individual level

Next, this paper investigates whether the impact of access to BPHSs on the settlement intent of migrants is heterogeneous at the individual level. By adding interaction items to the regression model, the heterogeneous effects among genders, educational levels and Hukou types are reported in Table 5. On the whole, although the significance level of some coefficients is low, the impact of establishing health records on migrants’ settlement intent seems greater for females, those who are less educated and those from the rural population. These results reflect how public health services protect the health rights of vulnerable groups and help promote their integration into society, thus encouraging them to settle in inflow areas.

**Table 5:** Heterogeneous effects at the individual level.

Variables	(1)	(2)	(3)	(4)	(5)	(6)
	Settlement intention	Settlement intention	Settlement intention	Settlement intention_per	Settlement intention_per	Settlement intention_per
Health record	0.0465***	0.0458***	0.0346***	0.0449***	0.0485***	0.0322***
	(0.0038)	(0.0040)	(0.0040)	(0.0049)	(0.0051)	(0.0064)
Male	0.0079***			-0.0206***		
	(0.0026)			(0.0032)		
Health record # Male	-0.0098**			-0.0040		
	(0.0040)			(0.0052)		
Educational level		0.0270***			0.1083***	
		(0.0030)			(0.0042)	
Health record # Educational level		-0.0091**			-0.0124*	
		(0.0045)			(0.0063)	
Hukou type			-0.0152***			-0.0736***
			(0.0034)			(0.0060)
Health record # Hukou type			0.0109**			0.0164**
			(0.0050)			(0.0071)
Controls	Yes	Yes	Yes	Yes	Yes	Yes
County FE	Yes	Yes	Yes	Yes	Yes	Yes
N	138,854	138,854	138,854	138,854	138,854	138,854
R-squared	0.0835	0.0835	0.0835	0.2439	0.2439	0.2439

Notes: This table reports the heterogeneous effect of establishing health records on migrants’ settlement intentions. Controls include all covariates in Table 2. Work industry information and work unit information are controlled in all columns. Standard errors in parentheses are clustered at the district level. Significance codes

*p<0.10

**p<0.05

***p<0.01.

### 3.5. Health-related knowledge and migrants’ settlement intentions

In addition to establishing health records, another important preventive care service is access to health-related knowledge, which is also provided by community services. In this section, the paper investigates whether health-related knowledge can improve migrants’ intent to settle. Table 6 reports the impact of access to knowledge on occupational diseases, infectious diseases, contraception and sterilization, chronic diseases, mental disorders, public emergencies and other aspects of health education on migrants’ settlement intentions. Although some coefficients have a low significance level, almost all kinds of health-related knowledge positively impact migrants’ settlement intentions. In particular, access to knowledge about contraception and sterilization, chronic diseases, mental disorders, and public emergencies has a large, significant impact on migrants’ settlement intent.

**Table 6:** Health-related knowledge and migrants’ settlement intentions.

Variables	(1)	(2)	(3)	(4)	(5)	(6)	(7)
	Occupational diseases	Infectious diseases	Contraception and sterilization	Chronic diseases	Mental disorders	Public emergencies	Other aspects
Panel A:							
Settlement intention	0.0033	0.0034	0.0101***	0.0075*	0.0109**	0.0106***	-0.0012
	(0.0034)	(0.0032)	(0.0037)	(0.0040)	(0.0047)	(0.0036)	(0.0035)
Panel B:							
Settlement intention_per	0.0061*	0.0070**	0.0257***	0.0237***	0.0223***	0.0079**	-0.0020
	(0.0035)	(0.0035)	(0.0036)	(0.0040)	(0.0043)	(0.0036)	(0.0044)

Notes: This table reports the impact of access to health-related knowledge on migrants’ settlement intentions. Individual and household characteristics, fixed district effects, work industry information and work unit information are controlled in all columns. The observations total 138,854 between all columns. The R-squared are 0.0814, 0.0814, 0.0815, 0.0814, 0.0815, 0.0815 and 0.0814 in Columns (1) to (7) in Panel A, respectively, and 0.2427, 0.2427, 0.2432, 0.2431, 0.2429, 0.2427 and 0.2426 in Panel B. Standard errors in parentheses are clustered at the district level. Significance codes

*p<0.10

**p<0.05

***p<0.01.

## 4. Conclusions

An important task in the urbanization process of developing countries is to promote and support migrants’ settlement in cities. Existing studies discussed how to improve migrants’ intent to settle from multiple perspectives, including human capital, social capital, household registration system, and social security, etc. Basic public health services (BPHSs) play an important role in improving the health status of migrant people and promoting social integration, yet existing studies have not focused on whether they can increase migrants’ settlement intentions.

Using data from the China Migrant Dynamics Survey (CMDS), this paper used the establishment of health records and access to health-related knowledge to examine the impact of BPHSs on migrants’ settlement intentions in China. The main results of this paper are: (1) establishing health records and providing access to health-related knowledge significantly positively impact migrants’ settlement intentions; (2) using instrumental variable regression to eliminate bias from the endogenous problem exemplifies the causal relationship between BPHSs and migrants’ settlement intentions; (3) BPHSs’ positive impact on migrants’ settlement intent is greater and more significant for female, less-educated and rural-Hukou migrants; (4) BPHSs positively impact migrants’ settlement intentions by improving their health and social integration.

This paper finds that basic public health services led by the government can improve the health and social integration of migrants during their urbanization, thus increasing their intent to settle permanently in inflow areas, with a more significant impact on vulnerable groups. Urbanization and citizenization are inevitable processes for developing countries to undergo, and the findings of this paper provide new factual evidence that may support government policymaking to further promote the integration of migrants. The government should recognize the important role of health services and medical resources in the process of improving the health status and quality of life of the migrant population. Therefore, to further promote urbanization, the government should focus on improving the quality and efficiency of basic public health services and increasing their coverage of them. In addition, the government should also improve the social medical security system through legislation and other means, especially to create convenient medical service conditions for vulnerable groups.
